# Recent Advances in the CRISPR/Cas-Based Nucleic Acid Biosensor for Food Analysis: A Review

**DOI:** 10.3390/foods13203222

**Published:** 2024-10-10

**Authors:** Yanan Sun, Tianjian Wen, Ping Zhang, Minglian Wang, Yuancong Xu

**Affiliations:** College of Chemistry and Life Science, Beijing University of Technology, Beijing 100124, China; syanan@emails.bjut.edu.cn (Y.S.); wtj_happy2005@163.com (T.W.); zplife@bjut.edu.cn (P.Z.); mlw@bjut.edu.cn (M.W.)

**Keywords:** CRISPR/Cas, biosensor, signal amplification, signal output, rapid detection, food analysis

## Abstract

Food safety is a major public health issue of global concern. In recent years, the CRISPR/Cas system has shown promise in the field of molecular detection. The system has been coupled with various nucleic acid amplification methods and combined with different signal output systems to develop a new generation of CRISPR/Cas-based nucleic acid biosensor technology. This review describes the design concept of the CRISPR/Cas-based nucleic acid biosensor and its application in food analysis. A detailed overview of different CRISPR/Cas systems, signal amplification methods, and signal output strategies is provided. CRISPR/Cas-based nucleic acid biosensors have the advantages of high sensitivity, strong specificity, and timeliness, achieving fast analysis of a variety of targets, including bacteria, toxins, metal ions, pesticides, veterinary drugs, and adulteration, promoting the development of rapid food safety detection technology. At the end, we also provide our outlook for the future development of CRISPR/Cas-based nucleic acid biosensors.

## 1. Introduction

Food is a basic condition for people’s survival, and food safety is directly related to the health and daily life of people. In recent years, food safety has become a worldwide public health concern, with contaminated food contributing to 600 million cases of foodborne illness and 420,000 deaths globally annually [1]. Microorganisms and toxins are now the typical risk factors for foodborne illnesses, in addition to pesticides, heavy metals, the adulteration of ingredients, genetic modification, illegal additives, etc., which also pose a threat to food safety [2]. Therefore, rapid and accurate testing can provide scientific and effective early warning for food safety incidents.

Testing techniques for these risk factors can be broadly categorized into two main groups: “traditional testing techniques” and “rapid testing techniques”. The traditional detection techniques used for microorganism detection are culture methods, and those used for pesticides and heavy metals are large-scale, instrumental, coupled methods. These traditional methods, while accurate, are time-consuming, highly specialized, and do not allow for real-time analysis. Common rapid detection technologies include immunoassay technology [3,4], nucleic acid amplification technology [5,6], colorimetric test strip technology [7,8], and biosensor technology [9,10], which effectively shorten the detection time, improve the specificity and sensitivity, and abandon the reliance on large-scale instrumentation, and they have become the mainstream technology for food safety. Among them, the nucleic acid-based biosensor can complete the recognition, amplification, and transduction of signals through nucleic acid intermediates and convert different targets into nucleic acids for analysis to achieve the normalized detection of different types of targets. Although the advantages are numerous, they are still limited by sample pretreatment, primer design, personnel expertise, and instrumentation.

**Figure 1:**
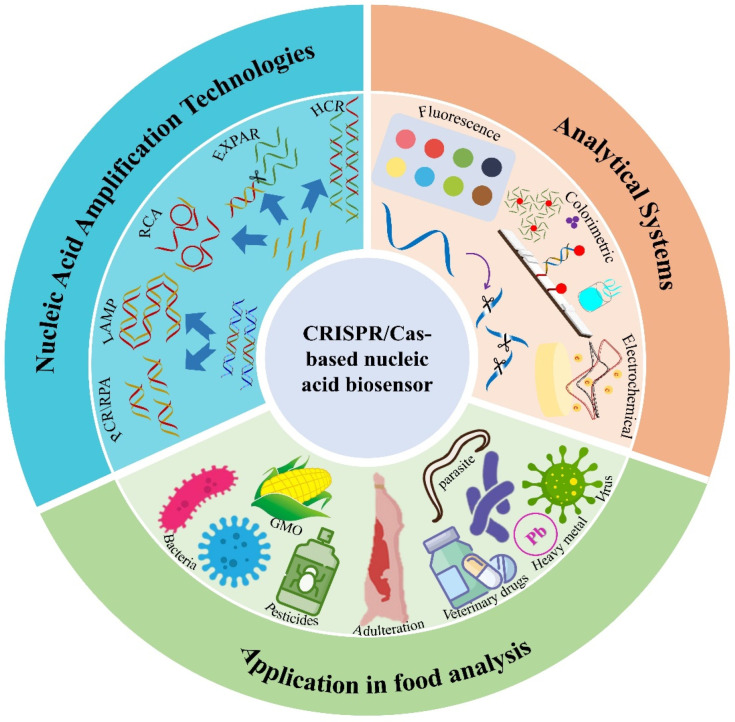
The CRISPR/Cas-based nucleic acid biosensor of food analysis. The blue part indicates nucleic acid amplification methods. The orange part indicates signal output analytical systems. The green part indicates food analysis targets.

The clustered regularly interspaced short palindromic repeats (CRISPR)/CRISPR-associated protein (Cas) (CRISPR/Cas) system is widely known for its excellent gene editing ability, unique target recognition method, and very high enzyme activity. It is an emerging molecular tool with a promising future in bioimaging, clinical therapeutics, infectious disease diagnostics, and environmental monitoring [11,12]. Different CRISPR systems have different Cas proteins, which can be flexibly selected for different target characteristics and have the advantages of high detection sensitivity and specificity, which is an important development direction in the field of rapid detection at present. The use of the CRISPR/Cas system in the construction of nucleic acid biosensors enables a perfect combination of the sensitivity, specificity, and flexibility of CRISPR/Cas with the simplicity, timeliness, and low cost of nucleic acid analysis. Through the integration of these new technologies, it is possible to quickly and accurately assess various risk factors in food, strengthen market supervision, and effectively stop problematic food from entering the market. In this review, we provide an overview of the research progress of CRISPR/Cas-based nucleic acid biosensors in the field of food analysis, analyzing the types of CRISPR/Cas systems, types of nucleic acid amplification combined methods, different readout strategies, and applications in different target assays (Figure 1). Through the analysis and comparison of different CRISPR/Cas-based nucleic acid biosensor detection platforms, future research directions and challenges in food analysis are prospected and discussed.

## 2. The CRISPR/Cas Systems

The CRISPR/Cas system is a kind of acquired immune system that exists in many archaea and bacteria [13]. According to the nature of Cas proteins, the systems can be divided into two categories. The Cas proteins of the class 1 systems are multi-subunit protein complexes whose activity exertion relies on the synergistic action of multiple effector proteins. The Cas proteins of the class 2 systems are independent proteins whose activity exertion only relies on a single effector protein [14]. Furthermore, according to gene structure and the structure of repeated spacer sequences, Cas proteins can be divided into six categories, namely, Cas protein I–VI. Cas proteins I, III, and IV belong to the class 1 system, while Cas proteins II, V, and VI belong to the class 2 system [15]. The mechanism of action of the CRISPR/Cas system consists of three steps, which are acquisition, expression, and interference. Firstly, the foreign gene is integrated between two repeats of the CRISPR locus to acquire interspaced sequences. Secondly, the locus is transcribed into precursor crRNA and then processed into mature crRNA (CRISPR RNA). Finally, crRNA is combined with Cas proteins to exert nuclease activity to cleave exogenous genes [16]. Based on the mechanism of action, the detection technology based on the CRISPR/Cas system designs single-guide RNA (sgRNA) according to detection target sequences. The cleavage activity of the Cas protein is activated after the sgRNA is recognized and paired with a specific target, and the detection of targets is achieved by analyzing the cleavage products. The cleavage activity includes *cis*-cleavage and *trans*-cleavage. *Cis*-cutting refers to the RNA-guided DNA cleavage activity and *trans*-cleavage refers to the nontarget ssDNA cleavage activity. Currently, CRISPR/Cas-based nucleic acid biosensor research mainly focuses on designing and applying four proteins, namely, Cas9, Cas12, Cas13, and Cas14 (Table 1). Cas9-based assays focus on the design of sgRNAs to achieve multiple assays through the specific recognition of target sequences, while Cas12-, Cas13-, and Cas14-based assays rely on the cleavage activity of the activated Cas protein.

### 2.1. CRISPR-Cas9

The Cas9 protein is the most typical type II CRISPR system effector protein. It is an RNA-guided and dsDNA (double-strand DNA)-targeted endonuclease. It contains two nuclease domains, namely, HNH and RuvC. HNH has only one nuclease region, while RuvC contains three nuclease regions. The CRISPR-Cas9 system requires Cas9, crRNA, and trans-activating RNA (tracrRNA) to act in collaboration. crRNA is combined with tracrRNA to construct a sgRNA. Target dsDNA containing a protospacer-adjacent motif (PAM) site (5′-NGG) can be recognized after Cas9 is partially bound to crRNA. crRNA and the complementary strand of target dsDNA form an RNA-DNA heteroduplex structure. Subsequently, the activity of the two nuclease structural domains of Cas9 is activated, which, in turn, cuts the target dsDNA [17]. By mutating the HNH and RuvC structural domains of Cas9, inactive dCas9 (nuclease-activated Cas9) proteins are obtained, which also recognize the target dsDNA but have no nuclease cleavage activity [18]. It was found that Cas9 can still exert cleavage activity on ssDNA targets after adding a small segment of PAMmer sequence alone to form a partial dsDNA-containing PAM sequence with the ssDNA target [19].

### 2.2. CRISPR-Cas 12

The Cas12 protein, a type V CRISPR system effector protein, is also an RNA-guided and DNA-targeted endonuclease and contains only the RuvC nuclease structure domain [20]. In the CRISPR-Cas12 system, the Cas12 protein can recognize the target dsDNA containing PAM site (5′-(T)TTN) under the guidance of crRNA or recognize the target ssDNA that does not require a PAM site to form a triple- or double-stranded complex. Subsequently, the nucleic acid endonuclease activity of RuvC is activated to specifically cleave target dsDNA or target ssDNA (cis-cleavage) and non-specifically cleave other ssDNA (*trans*-cleavage). However, one of the necessary conditions required for the activation of *trans*-cleavage activity is the requirement that at least 15 bases in the target sequence are complementary to the crRNA. Since the non-target strand (NTS) in the dsDNA target contributes to the stability of the “TS/crRNA/Cas12”, the dsDNA target activates a much higher *trans*-cleavage capacity than the ssDNA target [21]. A total of 14 different isoforms of the type V CRISPR system, Cas12a to Cas12n, have been identified to date. These proteins range in size from 400 to 1400 amino acids and show significant functional diversity [22]. Cas12a (Cpf1) and Cas12b (C2c1) are the most widely used in CRISPR biosensors, and the difference between the two is that the former does not require the involvement of tracrRNA for crRNA maturation, whereas the latter does, but both have endonuclease activity and *trans*-cleavage activity [23,24]. According to the mechanism of the CRISPR/Cas12a system, the Cas12a protein will first form a semi-closed R-loop with PAM recognition and then unwind the seed region to form a complete R-loop that furthers the allosteric activation of nuclease activity. Recent studies have found that PAM is only required for R-loop initiation, implying that artificial loop regions (pDNAs containing 6 nt bubbles) can also be used in place of PAM to enable the unwind and cleavage of dsDNA by Cas12a/crRNA complexes [25,26]. In addition, Cas12 family proteins normally recognize T-rich PAM, but Casπ (Cas12l) does recognize CCN PAM. Although the PAM sequences are different, the cis-cleavage and *trans*-cleavage activities of Casπ are comparable to those of Cas12a [27].

### 2.3. CRISPR-Cas 13

The Cas13 protein, a type VI CRISPR system effector protein, is an RNA-guided and RNA-targeted endonuclease that contains two HEPN nuclease domains [14]. In the CRISPR-Cas13 system, the Cas13 protein itself can process crRNA maturation without tracrRNA. Subsequently, under the guidance of crRNA, it can recognize the RNA-containing PFS (protospacer-flanking sequence) site (3′-A or U or C) and form a double-stranded RNA complex to promote HEPN1 and HEPN2 domains to get close to each other. Thus, it activates the endonuclease activity of HEPN nuclease to cleave specific target RNA and *trans*-cleavage activity to non-specifically cleave another on-target RNA. Among the family members of Cas13a-Cas13d for the Cas13 protein, there are many studies on Cas13a (C2c2), and different Cas13 proteins present different preferences for cleavage probes [28]. The nuclease-deactivated dCas13a obtained by mutating residues in the nuclease region binds to target RNA but has no cleavage activity [29].

### 2.4. CRISPR-Cas 14

The Cas14 protein, a type V CRISPR system effector protein, is an RNA-guided and ssDNA-targeted endonuclease that contains the RuvC nuclease domain. Cas14 belongs to the subclass of Cas12f, in which Cas14a, Cas14b, and Cas14c belong to Cas12f1, Cas12f2, and Cas12f3, respectively. CRISPR-Cas14 requires crRNA and tracrRNA to act in collaboration, but the recognition and cleavage do not require a PAM sequence. Cas14 specifically recognizes the target ssDNA and activates the endonuclease activity of RuvC to realize the cis- and *trans*-cleavage. Because of this, additional steps are required to convert dsDNA to ssDNA when testing for dsDNA. The advantage is that Cas14 is compact, half the size of Cas9, making it the smallest Cas protein [30]. The CRISPR/Cas14 system has a low tolerance for the mismatches between crRNA and ssDNA targets and successfully breaks through the shortage of accurate recognition of ssDNA targets, especially in the detection of single nucleotide polymorphisms, showing better specificity and accuracy, which marks huge progress for the CRISPR system [31].

## 3. Combination with Nucleic Acid Amplification Technologies (NAATs)

Different CRISPR/Cas systems recognize different types of nucleic acid targets. However, there are many problems such as low target concentration and matrix interference for the direct recognition in the CRISPR/Cas-based nucleic acid biosensor detection process. Generally, it is necessary to amplify the nucleic acid targets through NAAT and then establish a corresponding sensor platform by using the product as a new target combined with a CRISPR/Cas system. Different types of target products are obtained through different NAATs. The combination of the two can give full play to the detection function of the CRISPR/Cas system. Various CRISPR/Cas-based nucleic acid biosensors have been assembled and extensively applied in different detection fields. The differences and improvements between various biosensors mainly include five areas: (1) operating steps, such as the merging of amplification and analysis; (2) specificity, such as the identification of base mutations; (3) process contamination, such as closed-tube reactions to avoid cross-contamination; (4) detection throughput, such as the escalation of single- to multiplexed assays; and (5) result output, such as enabling naked-eye visualization. Most of these improvements rely on different NAATs. According to the degree of temperature dependence, NAATs can be divided into variable temperature amplification and isothermal amplification. According to the type of products, NAATs can be divided into double-stranded product amplification and single-stranded product amplification.

### 3.1. Polymerase Chain Reaction

Polymerase Chain Reaction (PCR), the most traditional NAAT, is a type of variable temperature amplification in which the product is dsDNA. HOLMES (one-HOur Low-cost Multipurpose highly Efficient System) is a classical platform based on CRISPR/Cas12a combined with PCR [32]. For 24 nt crRNA, HOLMES can recognize the PAM sequence and single base mutations at positions 1–7 after PAM, but it cannot distinguish the mutations at positions 8–18. For 16 nt and 17 nt crRNA, HOLMES can recognize base mutations at positions 1–16 after PAM. According to this characteristic, the specificity and sensitivity of SNP detection were enhanced by designing PCR primers with PAM but inserted near the SNP site. In the HOLMES detection platform, NAAT is not limited to PCR, and other amplification technologies can also be used. Ordinary PCR amplification takes about 2 h, while rapid PCR only needs 5 min. Wang et al. established CRISPCR (CRISPR and rapid PCR in a single capillary) by combining rapid PCR with CRISPR/Cas12a [33]. The rapid PCR reagent and CRISPR/Cas12a reagent were placed in the upper and lower parts of the PCR tube. The reagents were mixed for cleavage after amplification and the overall assay was completed in less than 10 min, being superior in terms of efficiency. Wang et al. combined multiplex PCR with CRISPR/Cas12a to establish a multiplex detection biosensor, achieving progression from single detection to high-throughput detection [34]. Different crRNA sequences were designed for different targets and the crRNAs were loaded onto different reaction wells to achieve five-fold detection by the separation system.

### 3.2. Loop-Mediated Isothermal Amplification

**Figure 2:**
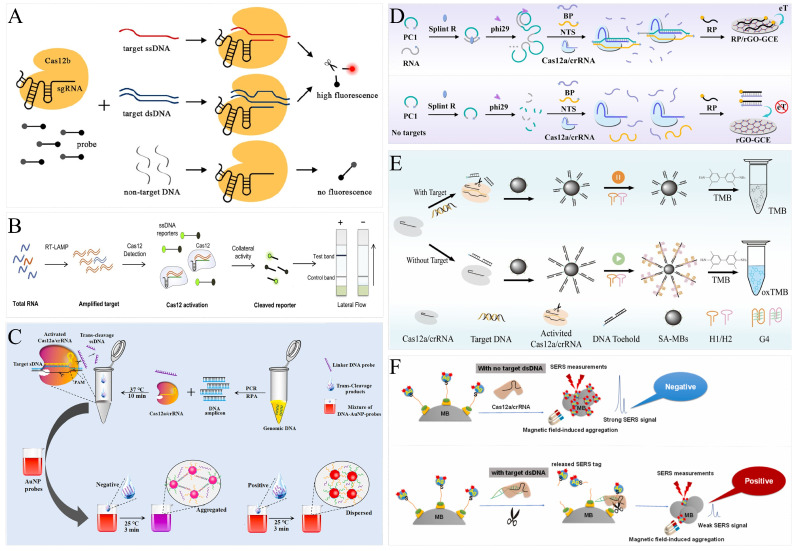
The principal diagram of signal amplification and signal output in the CRISPR/Cas-based nucleic acid biosensors. (**A**) Schematic illustration of fluorescence signal without nucleic acid amplification. Reprinted with permission from [35]. Copyright 2019 American Chemical Society. (**B**) Schematic illustration of LAMP combined with lateral flow strips [40]. Copyright 2020 Elsevier B.V. (**C**) Schematic illustration of PCR/RPA combined with AuNPs colorimetry. Reprinted with permission from [42]. Copyright 2022 Elsevier Ltd. (**D**) Schematic illustration of RCA combined with electrochemical signals. Reprinted with permission from [43]. Copyright 2021 American Chemical Society. (**E**) Schematic illustration of HCR combined with G4 colorimetry. Reprinted with permission from [44]. Copyright 2023 American Chemical Society. (**F**) Schematic illustration of SERS signal without nucleic acid amplification. Reprinted with permission from [45]. Copyright 2023 Elsevier B.V.

As a classical isothermal amplification technology, loop-mediated isothermal amplification (LAMP) can produce a lot of dsDNA products within a short time and does not depend on a thermal cycling apparatus. To simplify operation and avoid reaction cross-contamination, Li et al. established the HOLMESv2 platform based on HOLMES by combining CRISPR/Cas12b and LAMP again for the detection of DNA and RNA targets [35]. The fragments containing PAM sites were selected as the detection target in LAMP products and the *trans*-cleavage activity of Cas12a was activated after complementary pairing with crRNA (Figure 2A). Sam et al. established TB-QUICK (a novel M.tb DNA detection platform), which uses LAMP for nucleic acid amplification and detects mycobacterium tuberculosis based on the cis cleavage activity of CRISPR/Cas12b [36]. Zhang et al. designed a target-specific DNA hairpin probe to simplify LAMP and developed CAL-LAMP (CRISPR/Cas12a-Assisted Ligation-initiated LAMP) technology to detect ssDNA based on the *trans*-cleavage activity of CRISPR/Cas12a [37]. LAMP is widely used, but pollution has always been the biggest disadvantage, limiting the application of this technology. To avoid false positives caused by cross-contamination, Qian et al. established uracil-LAMP, replacing dTTP with dUTP, to obtain the amplification products containing dUTP [38]. At the same time, it was found that the PAM of Cas12b changed from “TTTN” to “UUUN” without affecting cleavage activities. Therefore, the ULC (UDG-LAMP-CRISPR) detection platform was established by combining uracil–DNA glycosylase (UDG), uracil–LAMP, and CRISPR/Cas12a. Firstly, enzymatic digestion was conducted with UDG to remove the remaining dUTP amplification products in the system and then amplification and subsequent cleavage reactions were conducted, which effectively avoided reaction contamination. The ULC method is effective but increases the complexity of the operation to some degree. Bao et al. established reliable and durable CRISPR/Cas9-based contamination-free loop-mediated isothermal amplification (CUT-LAMP) [39]. The core of CUT-LAMP is the use of PAM sequences to link two primers to design an inner primer. As a result, the LAMP products contained repeated PAM sequences and were cleaved and degraded under the recognition of Cas9/crRNA, thus avoiding contamination and effectively eliminating false positive reactions. LAMP technology is a detection method based on DNA amplification. For RNA targets, RT-LAMP needs to be established through reverse transcription and then combined with a CRISPR/Cas system. Ali et al. established the detection platform iSCAN (in vitro Specific CRISPR-based Assay for Nucleic acid detection), which combines RT-LAMP with CRISPR-Cas12 to detect RNA targets [40] (Figure 2B). Wang et al. developed the opvCRISPR (a one-pot visual reverse transcription (RT)-LAMP-CRISPR) system to detect SARS-CoV-2 in a single PCR tube by the systematic separation of RT-LAMP and Cas12a [41].

### 3.3. Recombinase Polymerase Amplification

Recombinase polymerase amplification (RPA) is an isothermal amplification technology that uses DNA as the template and relies on recombinase, single-stranded DNA-binding proteins (SSB) and DNA polymerase with strand-replacing activity; the amplification product is dsDNA (Figure 2C). SHERLOCK [46] and DETECTR [47] are two classic platforms of the CRISPR/Cas system combined with RPA.

The SHERLOCK (Specific High-Sensitivity EnzymaticReporter UnLOCKing) is a combination of CRISPR/Cas13a and RPA. For Cas13a, which recognizes RNA targets, it is necessary to transcribe RPA products (dsDNA) to RNA before it binds to crRNA and activates the *trans*-cleavage activity of Cas13a, which enables the differential detection of Zika, dengue viruses, pathogenic bacteria, and single-base mutations. Different Cas13 proteins have different preferences for cleavage probes. Based on this property, Gootenberg et al. further established the SHERLOCKv2 platform and achieved quadrupling detection by cleaving different probes through PsmCas13b, LwaCas13a, CcaCas13b, and Cas12a. The sensitivity was further enhanced by combining CRISPR-associated enzyme Csm6 [48]. Genome extraction of the target was required for the RPA. To simplify the nucleic acid extraction, Myhrvold et al. established HUDSON (Heating Unextracted Diagnostic Samples to Obliternucleases) platform by releasing nucleic acids through heat and chemical treatment and inactivating ribonuclease, further reducing detection times [49]. Arizti-Sanz et al. established the SHINE (SHERLOCK and HUDSON Integration to Navigate Epidemics) platform by combining SHERLOCK and HUDSON to further simplify the inspection system [50]. The specificity and sensitivity of the assay can be enhanced by RPA, but, because of the CRISPR/Cas13 system, an additional reverse transcription process is required, which increases the tediousness of the operation.

DETECTR (DNA Endonuclease-Targeted CRISPR Trans Reporter) is a combination of CRISPR/Cas12a and RPA. RPA products containing a PAM sequence bind to crRNA and activate the *trans*-cleavage activity of Cas12a to cleave the ssDNA probes. HPV16 and HPV18 are successfully detected by utilizing the differences in the last six bases of the “TTTC” PAM sequences. To simplify the reaction process and realize the integration of amplification and detection, one-pot RPA-CRISPR was developed. Wang et al. established the Cas12a-based Visual Detection (Cas12aVDet) platform by combining DETECTR and integrating all reagents into a PCR tube [51]. However, Cas12a was attached to the tube wall and mixed by centrifugation after RPA amplification to complete cleavage. Wang et al. developed a POCT-friendly detection method by combining RPA with CRISPR-Cas12a, which solved the compatibility of RPA and Cas12a cleavage [52]. All these methods successfully shortened the detection time from 2 h to 0.5 h, and the one-tube reaction effectively avoided cross-contamination.

Harrington et al. established the Cas14-DETECTR platform based on Cas14a and RPA [53]. For Cas14a, which recognizes ssDNA targets, it is necessary to transcribe RPA products (dsDNA) to ssDNA before it binds to crRNA. In the reaction, RPA amplification is first conducted by one phosphorothioate primer to generate single-labeled dsDNA products and then the unlabeled strand is hydrolyzed by T7 exonuclease to obtain the ssDNA product. Subsequently, it binds to crRNA and activates the *trans*-cleavage activity of Cas14a to cleave the ssDNA probes. Notably, Cas14 can detect very small DNA fragments compared to Cas12, making it particularly suited to high-resolution applications [54]. In addition to the classic combinations above, there are many applications of CRISPR/Cas systems combined with RPA, most of which aim to further improve specificity, sensitivity, and portability. For example, Teng et al. used RPA and CRISPR/Cas12b to develop a highly sensitive detection platform [55]; Xiong et al. used RT-RPA and CRISPR/Cas9 to detect viruses conveniently [56].

### 3.4. Nucleic Acid Sequence-Based Amplification

Nucleic Acid Sequence-Based Amplification (NASBA) is an isothermal amplification technology that uses RNA as the template and relies on avian myoblastosis virus (AMV) reverse transcriptase, Rnase H enzyme, and T7 RNA polymerase, in which the intermediate product is dsDNA. The NASBACC (NASBA/CRISPR cleavage) detection platform is the first application of the CRISPR/Cas system to be combined with NAAT [57]. Transcription after NASBA amplification yields RNA, which subsequently activates a color response of the sensor, enabling genotyping by differentiating between yellow and purple colors. The ingenuity of the method is that the PAM sequence selected for crRNA in the CRISPR/Cas9 happens to be genetically distinct fragments of the two Zika viruses. The dsDNA product containing “CGG” is obtained by NASBA amplification of the U. S. Zika. Then, it has a complementary pairing with crRNA and activates the cleavage activity of the Cas9 protein to cut off dsDNA. Only small segments of RNA are obtained after transcription, which fails to stimulate subsequent reactions and presents yellow results. By comparison, the African Zika has the base mutation, and the dsDNA product containing “CAG” is obtained after amplification. The cleavage activity of Cas9 is not activated because of the mutation of the PAM sequence. At this time, dsDNA can be transcribed to obtain complete RNA, and the subsequent biochemical reaction is activated, which presents purple results. In this way, the accurate identification and classification of Zika viruses are realized.

### 3.5. Rolling Circle Amplification

Rolling circle amplification (RCA) is one of the classical methods for single-stranded amplification, which can produce many consecutively repeated ssDNA products in a short time. This feature can be perfectly matched with CRISPR/Cas12 and CRISPR/Cas14a (Figure 2D). Ke et al. developed the RETA-CRISPR system for the accurate detection of circRNAs using RT-RCA and CRISPR/Cas12a [58]. Yang et al. developed a Cas14R system for high-specificity analysis of microRNA using RCA and CRISPR/Cas14 [59]. With the target as a primer, RCA amplification of the circle template containing the PAM sequence produces ssDNA products containing a lot of repeated target sequences, which bind to the crRNA and activate the *trans*-cleavage activity of Cas12 or Cas14a. For non-single-stranded nucleic acid targets, RCA amplification is also possible after single-stranded transformation. Xu et al. established a rapid detection method for bacteria based on RCA and CRISPR/Cas12a by using aptamer for target transduction [60]. Aptamers A and B of protein A on the surface of bacteria and PBPa2 protein are selected, in which aptamer A is coupled to magnetic beads and aptamer B is blocked by complementary chains. When target bacteria are mixed with the two aptamers, “aptamer A-bacteria—aptamer B” are separated through magnetic separation, and the remaining complementary chains are used for RCA. Then, the *trans*-cleavage activity of Cas12a is activated by the RCA products. Wang et al. combined RCA and CRISPR/Cas9 to establish the RACE (RCA-assisted CRISPR/Cas9 cleavage) detection platform [61]. After RCA, additional complementary probes are then added to assemble dsDNA products, which are recognized and paired with crRNA to activate Cas9 cleavage activity.

### 3.6. Strand Displacement Amplification

Strand displacement amplification (SDA) is a typical representative that relies on enzyme digestion reactions. Wang et al. achieved miRNA determination by coupling SDA and CRISPR/Cas12a [62]. During SDA, dsDNA is generated in the presence of strand-displacing enzymes and ssDNA in the presence of nick endonuclease, both of which can activate the *trans*-cleavage activity of Cas12. Wang et al. developed the TITAC-Cas (target-induced transcription amplification to trigger the *trans*-cleavage activity of Cas13a) detection platform, which is based on RNA polymerase-mediated SDA and CRISPR/Cas13a [63]. The T7 promoter sequence modified by the phosphate group forms a double-stranded substrate with the transcription template, and the T7 promoter sequence can be hydrolyzed by exonuclease. When ALP is present, the phosphate group is converted to a hydroxyl group, thus allowing the protection of the T7 promoter sequence, which, in turn, generates RNA in the presence of T7 RAN polymerase, which binds to the crRNA and activates the *trans*-cleavage activity of Cas13a. The exponential amplification reaction (EXPAR) is an SDA reaction amplified with single-stranded targets as primers. Huang et al. combined EXPAR and CRISPR/Cas9 to establish the CAS-EXPAR (CRISPR/Cas9 exponential amplification method) platform [64]. Unlike others, it utilizes CRISPR-stimulated EXPAR amplification. The ssDNA target is utilized to form ssDNA-dsDNA-Cas9/sgRNA complexes containing PAM sequences to activate the cleavage activity of Cas9, and the formed product is used as a primer to trigger EXPAR.

### 3.7. Hybridization Chain Reaction

Hybridization chain reaction (HCR) is a typical enzyme-free amplification technique achieved by the self-assembly of a single-stranded nucleic acid and two hairpin nucleic acids, generating a long-nicked DNA duplex. With the advantages of high sensitivity, flexible structure, low cost, and anti-contamination properties, HCR has been widely used in biosensing platforms (Figure 2E). Jia et al. developed a fluorescence biosensing platform based on the CRISPR/Cas12a system combined with HCR [65]. The target RNA triggers HCR to produce a large dsDNA product, which recognizes the crRNA and activates the *trans*-cleavage activity of Cas12a to cleave the ssDNA probe. Wang et al. developed a colorimetric biosensor based on the CRISPR/Cas12a system combined with aptamer and HCR [66]. After recognizing the target, the aptamer releases the complementary strand and acts as promoter DNA to trigger the downstream HCR. Subsequently, the *trans*-cleavage activity of Cas12a is activated, which cuts the ssDNA linker, leading to the dispersion of the DNA-AuNPs probe and a red coloration of the solution. In addition to the amplification followed by cleavage mode, there is also cleavage followed by the amplification mode. Ke et al. proposed a CRISPR/Cas12a-based chemiluminescence enhancement biosensor (CLE-CRISPR) based on HCR [67]. The ssDNA probe was designed as an intermediate to release promoter DNA by strand displacement, which, in turn, triggers HCR. By introducing Bio-H2, long double-stranded DNA frameworks containing many biotins can be generated to capture the streptavidin–AP generation signal. In the presence of target nucleic acids, activated CRISPR/Cas12a is able to randomly cleave the ssDNA probe into short fragments, and then no subsequent HCR can be triggered. As a result, no signal is generated. Similarly, Yang et al. developed a CRISPR-based evanescent wave fluorescence biosensing platform based on the dual-labeled HCR [68]. By recognizing the target RNA and activating Cas13a activity, the hairpin DNA probe is cleaved, releasing promoter DNA and triggering the downstream HCR. The Bio-dsDNA-Cy5.5 products are generated by introducing Bio-H1 and Cy5.5-H2. The products are immobilized on the surface of DTB-functionalized fibers by biotin–streptavidin, thus enabling the immobilization of Cy5.5. As a result, strong fluorescent signals are generated.

## 4. CRISPR/Cas Analytical Systems

Nucleic acid amplification can effectively amplify a target concentration and then activate the cleavage activity of Cas proteins. Activated Cas proteins shear target nucleic acids and random ssDNA probes like scissors and output a variety of measurable signals. Typically, three types of signals can be obtained by combining different tags. (1) Fluorescence. Upon cutting, the quenching group separates from the fluorescent group, resulting in an enhanced fluorescence signal. The change in fluorescence can be observed by the naked eye under UV light and quantified by a spectrofluorimeter. (2) Colorimetric. Color changes are caused by physical changes in nanomaterials and chemical changes in special substrates. The color change can be directly observed visually and quantified by a visible spectrophotometer. (3) Electrochemical. Current changes are caused by chemical transformations like oxidation or reductions on the surface of an electrode, which involve electron transfer. Quantitative analysis can be realized by current changes. Fluorescence and colorimetric are the two most widely used analytical methods.

### 4.1. Fluorescence Analytical Systems

Fluorescence is the most classic analytical system for CRISPR/Cas-based biosensors (Figure 2A). The most important advantages are low background interference and high sensitivity. The classic SHERLOCK, HOLMES, and DETECTR all enable target analysis via fluorescence signals. In most analyses, Cas proteins exert *trans*-cleavage activity that cleaves the fluorophore–quencher-labeled probe, allowing fluorescence to be restored by separation of the fluorophore and quencher [69,70]. Niu et al. used spatial conformational changes of aptamers to alter Cas protein activity for small molecule detection [71]. Aptamers could bind to Cas 12a/crRNA and activate *trans*-cleavage activity, resulting in changes in fluorescence intensity. In the presence of target material, it could bind to the aptamer and change the spatial conformation of the aptamer. As a result, it could not bind to crRNA and, thus, there was no *trans*-cleavage. The concentration of the target substance in the system was inversely proportional to the fluorescence intensity, and the sensitive detection of small molecular targets could be realized through the fluorescence intensity. Sha et al. accomplished the ultra-sensitive and direct detection of microRNA by connecting CRISPR/Cas13a and CRISPR/Cas14a [72]. The *trans*-cleavage activity of Cas13a was utilized to cleave the single-stranded ring substrate, followed by excitation of the *trans*-cleavage activity of Cas14a, which cleaved the fluorescence-quenched probe in the system, and the detection was accomplished by the change in fluorescence. Choi et al. achieved fluorescence analysis using nanogold instead of a quencher [73]. A 7 nm long ssDNA fluorescent probe was connected to a 20 nm gold particle and a 9 nm long ssDNA probe was connected to a 60 nm gold particle. The fluorescence of the two probes was quenched by the 60 nm gold particle after they were assembled by base pairing. crRNA was designed according to the nucleic acid target, and the activity of Cas12a could not be activated in the absence of the target. When the single-stranded nucleic acid target existed, it could bind to crRNA/Cas 12a and activate the cleavage activity, cutting off the single-stranded part of the probe and resulting in fluorescence recovery. The 20 nm gold particles could further enhance fluorescence. The effective and sensitive detection of cancer nucleic acid marker targets could be realized through the change in fluorescence.

### 4.2. Colorimetric Analytical Systems

AuNPs are an attractive material in the field of colorimetric analysis. Monodisperse AuNPs are red in color, while aggregated AuNPs are blue. Zhou et al. used this property to design a AuNP bioprobe and DNA linker [42]. The complementary hybridization of AuNP probes with DNA linkers led to purple AuNP aggregates. When the *trans*-cleavage activity of CAas12a was activated, it cleaved the DNA linker, at which point the AuNP dispersion appeared red (Figure 2C). Chen et al. constructed a MAPs nanoprobe consisting of magnetic beads, DNA, and AuNP [74]. When Cas14a was activated, the MAPs nanoprobes were cleaved, releasing free AuNP, which could not be removed even by magnetic separation, and the system underwent a change from colorless to red. Xu et al. developed RPA-CRISPR/Cas12a-LFS for nucleic acid visualization analysis [75]. The ssDNA probe hybridized to AuNP-AP to form the LP/AuNP-AP complex, which could be captured by the test line of the strip. The ssDNA probe was cleaved when the RPA product activated the *trans*-cleavage activity of CRISPR/Cas12a. AuNP would not appear on the test line of the strip, and no red band appeared. Wang et al. developed a CRISPR/Cas9-mediated lateral flow strip based on the silver-coated gold nanostar (AuNS@Ag) [76]. When the biotinylated target gene was recognized and unwounded by the CRISPR/Cas9 system, the AuNS@Ag probe was able to hybridize with the NTS. This complex was then captured by the test line of the strip. Red bands appeared on the test line due to the aggregation of AuNP.

In addition to the agglomeration effect of nanomaterials, there are also chemical reactions that can cause color changes. For example, horseradish peroxidase catalyzes a significant color change in 3,3′,5,5′-tetramethylbenzidine (TMB) and 2,20-azino-bis (3-ethylbenzthiazo-line- 6-sulfonic acid) diammonium salt (ABTS). Jiang et al. developed a colorimetric assay based on a hybridization chain reaction and CRISPR/Cas12a [44]. The magnetic bead probe was able to hybridize two hairpins to trigger a hybridization chain reaction, generating a G-rich single-stranded product. After folding to form a hemin/G-quadruplex, it exerted peroxidase-like activity and catalyzed the color change of the TMB. Once the *trans*-cleavage activity of CRISPR/Cas12a was activated, it cleaved the magnetic bead probe, resulting in HCR failure, with no change in color (Figure 2E). Xu et al. designed a MnO_2_-ssDNA-MBs probe to achieve colorimetric analysis [77]. The activated Cas12a cleaved the probe, separating MnO_2_ from the surface of MBs to the supernatant. Then, MnO_2_ nanozymes exerted peroxidase-like activity to induce the color change of TMB. Similarly, Gong et al. designed a GO-ssDNA-MBs probe to achieve colorimetric analysis [78]. The released GOx catalyzed color changes in TMB. In addition, Zhao et al. developed conjugation-free CRISPR/Cas12a-responsive hydrogels for colorimetric analysis [79]. DNA hydrogels wrapped with amylase (GA) were prepared using RCA. When the *trans*-cleavage activity of Cas12a was activated, the DNA hydrogel was crushed to release GA. GA was able to break down the amylum strains, thereby changing the amylum-I2 mixture from dark blue to colorless.

### 4.3. Electrochemical Analytical Systems

Electrochemical biosensors, with their advantages of high sensitivity, fast signal readout, and low background values, have become another alternative analytical platform. Li et al. introduced an electrochemical biosensor that combined recombinase-aid amplification (RAA) and CRISPR/Cas12a to detect bacteria [80]. A methylene blue (MB)-labeled ssDNA reporter was assembled on the surface of the working electrode. The transfer of electrons from the MB to the electrode produced a large current. The ssDNA was degraded by activated Cas12a, which led to the separation of MB from the electrode, thus inhibiting the electron transfer and decreasing the output current. Suea-Ngam et al. presented a silver-enhanced electrochemical CRISPR/Cas biosensor to detect bacteria [81]. Silver could be deposited on the ssDNA on the electrode surface, thus generating a strong current signal. Once the *trans*-cleavage activity of Cas12a was initiated, it cleaved the ssDNA on the electrode surface, preventing the subsequent deposition of silver and thus lowering the current. Qing et al. developed an immobilization-free electrochemical biosensing platform by combining RCA with CRISPR/Cas12a [43]. The RCA product hybridized to crRNA and activated the *trans*-cleavage activity of Cas12a to cleave the ssDNA probe, which was complementary to the MB-labeled probe. In the subsequent analysis system, the newly added MB-labeled ssDNA probe could be adsorbed by a reduced graphene oxide-modified electrode (rGO/GCE), resulting in an obvious electrochemical signal. On the contrary, the uncleaved ssDNA probe hybridized with the MB-labeled ssDNA probe to form dsDNA that could not be captured by the rGO/GCE, resulting in a negligible electrochemical signal (Figure 2D). Liu et al. established a label-free electrochemical biosensor based on the electrostatic adsorption of Ru(NH_3_)_6_^3+^ [82]. The gold electrode was modified with ssDNA probes that could initiate the hybridization chain reaction with two hairpins. Subsequently, a large amount of dsDNA was assembled on the electrode surface. The positively charged Ru(NH_3_)_6_^3+^ was then bound to the dsDNA by an electrostatic interaction, generating a high differential pulse voltammetry (DPV) signal. Once the *trans*-cleavage activity of Cas12a was triggered, the formation of the dsDNA products was prevented, which, in turn, prevented the loading of Ru(NH_3_)_6_^3+^ onto the electrode, resulting in a rather low DPV signal.

### 4.4. Other Analytical Systems

In addition to fluorescent groups, nanomaterials, and electroactive molecules, ssDNA probes can also be labeled with other substances to produce other types of output signals. Lin et al. introduced a CRISPR-mediated luminescence resonance energy transfer (LRET)-based aptasensor using upconversion nanoparticles (UCNPs) as luminescence labels and MXene-Au nanohybrids as an enhanced quencher [83]. The ssDNA-UCNPs could be adsorbed onto the surface of MXene-Au via a hydrogen and chelation interaction, bringing UCNPs close to MXene-Au and thus greatly reducing the upconversion luminescence (UCL) via LRET. When the aptamer was hybridized to crRNA and activated the *trans*-cleavage activity of Cas12a, the ssDNA-UCNPs were cleaved and the UCNPs were unable to be adsorbed onto the MXene-Au, and the UCL remained. Wang et al. developed a CRISPR-mediated surface enhancement of Raman scattering (SERS) biosensor using 4-MBA as a Raman reporter [45]. The core of the design was the AgNPs/4-MBA/ssDNA/MBs SERS probe, which generated a strong SERS signal. Once Cas12a was activated, 4-MBA left the MB and produced a weak SERS signal (Figure 2F). The silicon microring resonator (SMR) biosensors were analyzed based on changes in the refractive index near the waveguide surface. Koo et al. established a CRISPR/dCas9-mediated diagnostic tool based on SMR biosensors [84]. The specific primers were immobilized as probes on the surface of the SMR biosensor. After amplification, dCas9 bound to the amplification product, increasing the molecular weight of the SMR probes, which, in turn, increased the refractive index change to improve the detection signal.

## 5. Applications in Rapid Food Analysis

Nucleic acid biosensors convert target signals to nucleic acid signals and then to chemical signals via nucleic acids. The CRISPR/Cas nucleic acid biosensor, on the other hand, introduces the CRISPR/Cas system, which has made waves in the detection field, mainly by means of the *trans*-cleavage activity of Cas proteins. A number of detection platforms and methods have been developed utilizing Cas proteins, such as SHERLOCK [46] and DETECTR [47]. The development of these platforms reduces the requirements for instruments and personnel in detection and provides new ideas for food detection. Therefore, various CRISPR detection platforms that combine different Cas proteins, signal amplification methods, and signal analytical systems have been used to develop various platforms for food safety detection. This CRISPR/Cas-based nucleic acid biosensor, as a coarse screening tool for food analysis, can significantly improve analytical efficiency and is important for risk assessment. Currently, CRISPR/Cas-based nucleic acid biosensors are gradually being applied to the rapid detection of nucleic acid-based and non-nucleic acid-based risk factors (Table 2).

### 5.1. Detection of Foodborne Pathogens

Food safety incidents caused by foodborne pathogens have accounted for a large proportion of health incidents for a long period. There are three ways to construct CRISPR/Cas-based nucleic acid biosensors for pathogen detection. In the first, the biosensor is established by genome extraction, nucleic acid amplification, and the CRISPR/Cas system. In the second, the biosensor is constructed by aptamer transformation and the CRISPR/Cas system. In the third, the biosensor is created by aptamer transformation, nucleic acid amplification, and the CRISPR/Cas system. Among them, the latter two methods can avoid the tedious process of bacterial culture and genome extraction.

**Figure 3:**
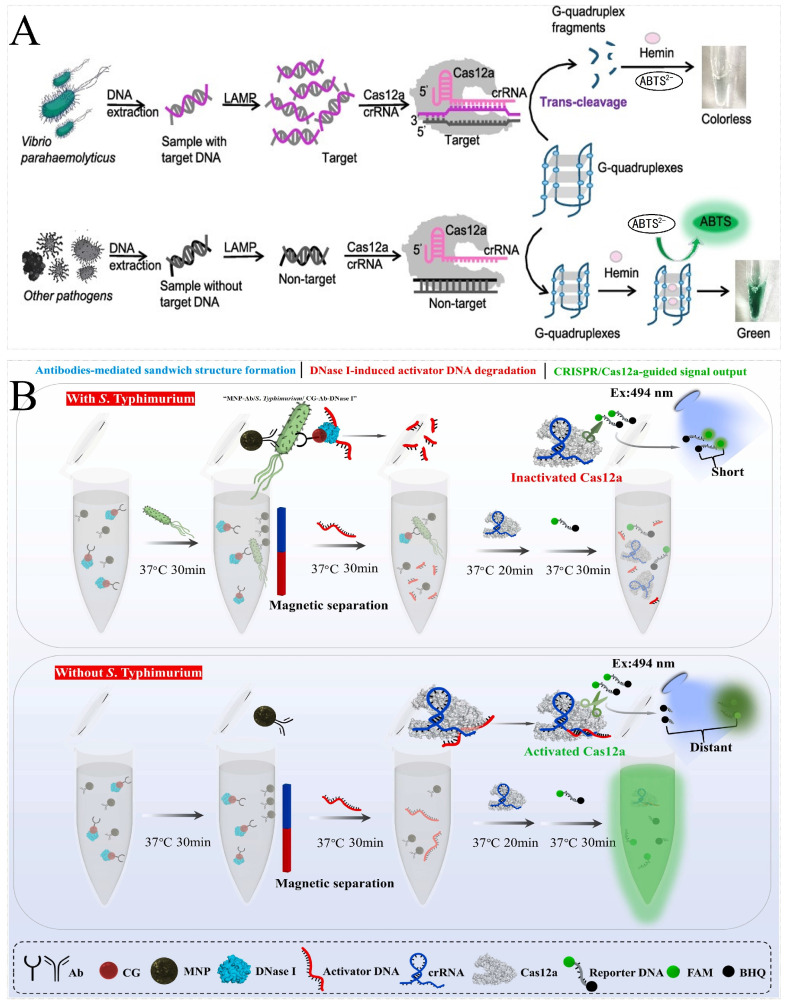
Principal diagram of foodborne pathogen detection by CRISPR/Cas-based nucleic acid biosensors. (**A**) Schematic illustration of the label-free colorimetric biosensor for the detection of *Vibrio parahaemolyticus*. In the presence of *Vibrio parahaemolyticus*, LAMP products combine with crRNA. The active Cas12a cleaves the G4 probe and is unable to catalyze a color change in ABTS^2−^. The positive and negative results can then be easily distinguished by the obvious color differences. Reprinted with permission from [87]. Copyright 2021 American Chemical Society. (**B**) Schematic illustration of the CATCHER for the detection of *Salmonella Typhimurium*. Once the “MNP-Ab/*S. Typhimurium*/CG-Ab-DNase I” is formed, DNase I converts activator DNA to invalid DNA fragments, resulting in the inactivation of Cas12a and the silencing of the signal output system. Reprinted with permission from [90]. Copyright 2022 Elsevier B.V.

Peng et al. combined the CRISPR/Cas12a system and the PCR amplification method to develop a fluorescence biosensor for *Staphylococcus aureus* [85]. Zhou et al. combined the CRISPR/Cas12a system and the RAA (with the same principle as RPA) amplification method with QDs (quantum dots) to develop a lateral flow biosensor for *Staphylococcus aureus*. The degrading probe failed to capture the QD-SA to intercept on the T line and led to no signal accumulation. Due to the accumulation of QDs, the signal at the lateral flow biosensor could be detected by the naked eye and a fluorescence reader [86]. Chen et al. combined the CRISPR/Cas12a system and the LAMP amplification method with G-quadruplex DNAzyme to develop a label-free colorimetric biosensor for *Vibrio parahaemolyticus*. G-quadruplex DNAzyme (G4) can fold to form a G-quadruplex structure, which binds hemin to exert peroxidase-like activity and catalyzes ABTS^2−^, which turns green. The positive and negative results can then be easily distinguished by the obvious color differences. This method can detect 9.8 CFU/reaction and 6.1 × 10^2^ CFU/mL of pure cultured and spiked samples, respectively (Figure 3A) [87]. Multiple testing can significantly improve detection efficiency. Gootenberg et al. combined the CRISPR/Cas13 system and the multiple RPA amplification method to develop a dual CRISPR/Cas fluorescence biosensor to simultaneously detect *Staphylococcus aureus* and *Pseudomonas aeruginosa*. Following the preference of Cas13 for the cleavage probe, the poly U probe and poly A probe were cleaved by Cas13a and Cas13b, respectively. The *Staphylococcus aureus* and *Pseudomonas aeruginosa* were simultaneously detected by changing the intensity of two kinds of fluorescence [48].

It is important to note that most of the described assays require nucleic acid amplification, which can easily lead to false positives due to contamination. Li et al. developed a CRISPR/Cas14a biosensor without nucleic acid amplification. The PtPd@PCN-224 nanoenzymes were prepared by growing PtPd nanoparticles on the surface of PCN-224 nanorods, which were able to bind to P-ssDNA and be immobilized at the electrodes via Zr-O-P ligand bonds. It had excellent peroxidase-like activity and catalyzed H_2_O_2_ degradation at the electrode surface to trigger current changes. A PtPd@PCN-224 nanozyme coupled CRISPR electrochemical biosensor for the detection of *Burkholderia pseudomallei* was reported, which had a detection limit as low as 12.8 aM for *Burkholderia pseudomallei* DNA [88]. However, all these methods relied on the genome DNA, but DNA extraction is problematic for some non-culturable or difficult-to-cultivate bacteria. Duan et al. combined the CRISPR/Cas12a system with aptamer to develop a CATCHER biosensor for the detection of *Salmonella Typhimurium*, which has a detection limit of 7.9 × 10^1^ CFU/mL in PBS and 6.31 × 10^3^ CFU/mL in spiked samples, respectively (Figure 3B) [90].

### 5.2. Detection of Biotech Foods

Biotech foods generally refer to the food obtained based on biotechnology. The main ones that people are more familiar with now are genetically modified organisms (GMOs). Generally, three steps are required to analyze GMOs using a CRISPR/Cas-based nucleic acid biosensor: genome extraction, nucleic acid amplification, and *trans*-cleavage. Liu et al. developed an RPA-Cas12a-FS fluorescence biosensor to detect transgenic products. This method can specifically detect target gene levels down to 10 copies in 45 min without specialized techniques, which holds good promise for the rapid on-site detection of transgenic products [91]. Pataer et al. developed a multiplex LAMP-based CRISPR/Cas12a method for the sensitive detection of GMOs, which had a detection limit of 100 aM. The triple detection mode significantly improved detection sensitivity compared to stand-alone detection [94]. For deeply processed foods, the genome was disrupted and there were more short segments. Peng et al. used a multiple-CRISPR-derived RNA (crRNA) system to detect short DNA in processed GMOs. Three crRNAs were designed to detect three nucleic acid fragments. This method avoided possible aerosol contamination from nucleic acid amplification while saving assay time [93].

**Figure 4:**
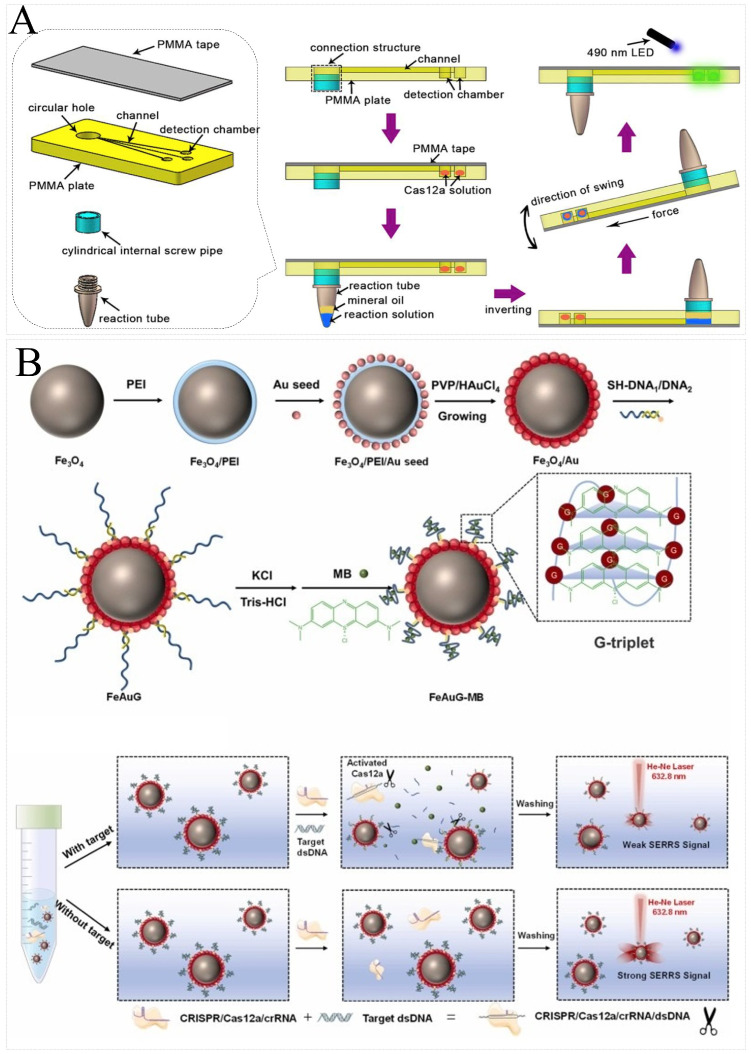
Principal diagram of biotech food detection by CRISPR/Cas-based nucleic acid biosensors. (**A**) Schematic illustration of Cas12a-PB for the detection of GM soybeans. LAMP assay reagents and CRISPR/Cas12a assay reagents were preloaded onto PCR tubes and detection chambers of PMMA, respectively. After amplification, the reaction tube was inverted and the detection was completed by fluorescence changes in different detection chambers. Reprinted with permission from [95]. Copyright 2020 Elsevier B.V. (**B**) Schematic illustration of the SERRS assay for the identification of GMOs. A magnetic SERRS nanoprobe (FeAuG-MB) was constructed by modifying the G-quadruplex on the surface of gold-coated magnetic beads. Methylene blue (MB) was inserted into the G-quadruplex as a SERS reporter molecule. When the *trans*-cleavage of the CRISPR/Cas12a was activated, the G-triplet DNA was cleaved, which caused a release of methylene blue above the nanoprobes and a reduction in the SERRS signal. Reprinted with permission from [92]. Copyright 2024 Elsevier B.V.

To improve portability, Wu et al. developed a CRISPR/Cas12a-based portable biosensor (Cas12a-PB) to detect GM soybeans. Emphasis was placed on the preparation of polymethylmethacrylate (PMMA) with a connection structure, three channels, and three detection chambers. LAMP assay reagents and CRISPR/Cas12a assay reagents were preloaded onto PCR tubes and detection chambers, respectively. This novel portable dual detection platform successfully realized the simultaneous detection of the CaMV35S promoter and lectin gene, with a detection limit of 0.1% (Figure 4A) [95]. CRISPR/Cas12a was coupled with SERS to form a variety of novel platforms for the detection of GMOs. Su et al. combined CRISPR and SERS with a magnetic SERRS nanoprobe (FeAuG-MB) to convert the detection of GMOs’ DNA into a SERS signal and applied it to the ultrasensitive detection of 35S and Nos genes, showing excellent specificity and sensitivity (Figure 4B) [92].

### 5.3. Detection of Food Adulteration

**Figure 5:**
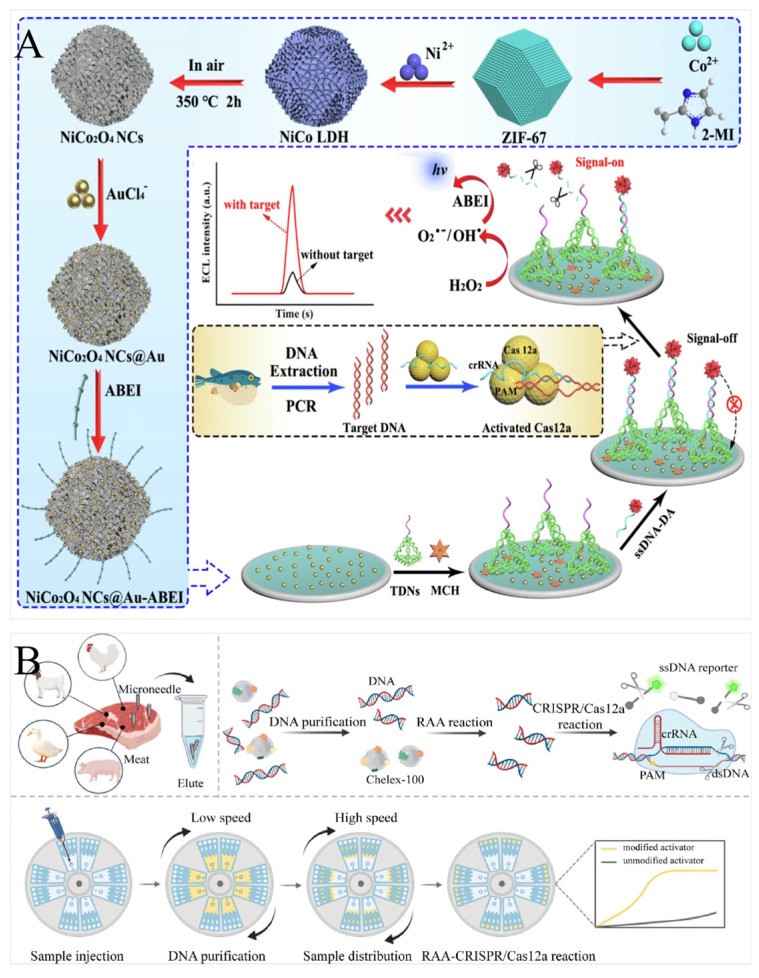
Principal diagram of food adulteration detection by CRISPR/Cas-based nucleic acid biosensors. (**A**) Schematic illustration of the ECL biosensor for the detection of pufferfish. This involved NiCo_2_O_4_ NCs@Au-ABEI designed as nanoemitters that were immobilized on the electrode surface by nucleic acid hybridization. The *trans*-cleavage activity of CRISPR/Cas12a was initiated when pufferfish DNA was present, leading to a nonspecifically cleaved ssDNA and releasing NiCo_2_O_4_ NCs@Au-ABEI. As a result, the ECL signal was significantly increased. Reprinted with permission from [98]. Copyright 2024 Elsevier Ltd. (**B**) Schematic illustration of CIPAM for the simultaneous detection of pig, chicken, duck, and lamb. The modules of the centrifugal microfluidic chip were pre-loaded with the reagents required for the RAA-CRISPR/Cas12a reaction for integrated operation. If target DNA was present, a fluorescent signal appeared in the corresponding reaction module. Reprinted with permission from [100]. Copyright 2024 Elsevier Ltd.

Currently, the incidence of food adulteration is increasing globally and there is an urgent need to develop robust techniques for the rapid analysis of food adulteration. To address the difficult-to-detect problem of food adulteration, several novel platforms have been developed, in which the cas12a protein plays an important role. CRISPR/Cas12a-based nucleic acid biosensor detection also requires three steps, namely, genome extraction, nucleic acid amplification, and Cas cleavage. Liu et al. designed an RPA-CRISPR/Cas12a-LFD system for the field detection of meat. The results showed that the meat samples could be detected with high specificity, and the LOD was as low as 1 × 100 copies/μL, demonstrating high selectivity [96]. Pan et al. proposed a CRISPR/Cas12a-based SERS biosensor for goat milk authenticity detection, which had a minimum goar DNA concentration of 224 aM [97]. In addition to detecting pufferfish DNA, Zhang et al. combined the PCR-CRISPR/Cas12a system with NiCo_2_O_4_ NCs@Au-ABEI nanoemitters to develop a novel electrochemiluminescence (ECL) biosensor to identify pufferfish authenticity with a detection limit of 0.1% (*w*/*w*) (Figure 5A) [98].

To improve detection throughput, Xiang et al. combined a centrifugal microfluidic chip with the RAA-CRISPR/Cas12a system to develop a novel detection platform, centrifugal integrated purification–CRISPR array (CIPAM), applied to meat detection within 30 min. CIPAM had excellent sensitivity with detection limits as low as 0.1% (*w*/*w*) for pig, chicken, duck, and lamb products, with outstanding specificity in the analysis of real samples (Figure 5B) [100]. However, genome extraction is often required for such biological adulteration analyses. To simplify genome extraction, Zhao et al. used alkaline lysis to extract DNA within 22 min. Then, an RPA-CRISPR/Cas12a assay was developed to detect pork DNA. All process steps could be completed in 30 min with a detection limit as low as 10^−3^ ng, demonstrating excellent specificity and sensitivity [99].

### 5.4. Detection of Biotoxins

Biotoxins are poisonous and harmful substances produced by animals, plants, and microorganisms. Most food safety incidents are caused by toxins secreted by microorganisms. Toxins are non-nucleic acid substances, so aptamers are mostly needed for nucleic acid transformation when using a CRISPR/Cas nucleic acid sensor for detection. The *trans*-cleavage strategy plays an important role in CRISPR/Cas-based toxin detection. Mao et al. designed a CRISPR/Cas12a-based aptasensor based on an upconversion-magnetic probe–DNA-Fe_3_O_4_ probe to detect OTA, which had a detection limit of 1.5641 ng/mL [102]. To further increase sensitivity, Chen et al. developed a novel CRISPR/Cas12a enzyme-linked aptamer–sorbent assay (CLASA) platform and applied it to OTA analysis with a detection limit of 171 fg/mL [101].

**Figure 6:**
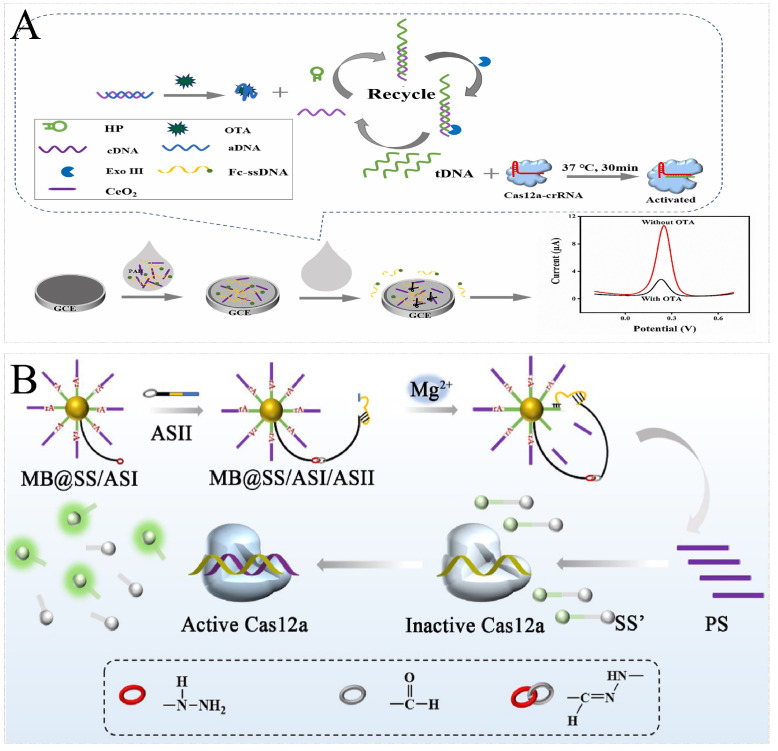
Principal diagram of biotoxin detection by CRISPR/Cas-based nucleic acid biosensors. (**A**) Schematic illustration of the neoteric electrochemical biosensor for the detection of OTA. Using an Exo III-assisted recirculation reaction, the target recognition transaction was converted into a triggering DNA strand and amplified into the *trans*-cleavage activity of Cas12a to detect changes in electrochemical signals. Reprinted with permission from [103]. Copyright 2023 Elsevier B.V. (**B**) Schematic illustration of the hydrazone ligation-assisted DNAzyme walking nanomachine for the detection of lipopolysaccharide. In the presence of LPS, LPS bound to the aptamer in the complex to release ASII, triggering subsequent reactions and enhancing fluorescence. Reprinted with permission from [104]. Copyright 2021 Elsevier B.V.

In addition, with the assistance of nucleic acid amplification, ultrasensitive detection can be further achieved. Wu et al. introduced a neoteric CRISPR/Cas12a electrochemical biosensor based on exonuclease III-assisted signal amplification to detect OTA with a detection limit of 0.74 fg/mL (Figure 6A) [103]. Similarly, Wang et al. combined the CRISPR/Cas12a system with cascade signal amplification to develop a hydrazone ligation-assisted DNAzyme walking nanomachine applied to the highly sensitive detection of LPS, which could sensitively identify LPS in spiked soft drink samples in the range of 0.05–106 ng/mL, with a detection limit of 7.31 fg/mL (Figure 6B) [104]. In addition to signal transduction properties, nanomaterials can also be applied to detection to improve sensitivity. Abnous et al. reported an assay platform combining CRISPR/Cas12a with RCA and AuNPs applied to the detection of AFM1, with a detection limit as low as 0.05 ng/L [105].

### 5.5. Detection of Heavy Metal Ions

**Figure 7:**
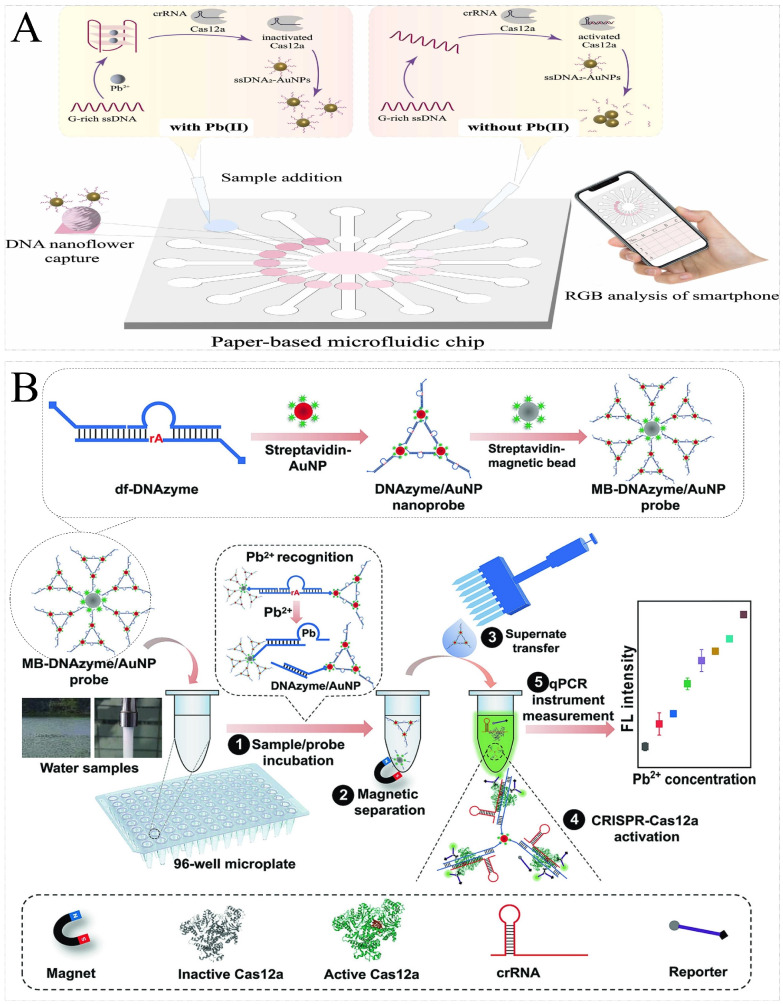
Principal diagram of heavy metal ion detection by CRISPR/Cas-based nucleic acid biosensors. (**A**) Schematic illustration of the dual-functional DNAzyme-powered CRISPR-Cas12a biosensor for the detection of Pb^2+^. In the presence of Pb^2+^, the DNAzyme was cleaved, causing disintegration of the MB-DNAzyme/AuNP probe. The degraded DNAzyme/AuNP in the supernatant effectively triggered CRISPR/Cas12a activity, initiating DNA reporter cleavage and generating great fluorescence. Reprinted with permission from [108]. Copyright 2023 Elsevier B.V. (**B**) Schematic illustration of the paper-based microfluidic biosensor for the detection of Pb^2+^. The Pb^2+^-induced G-quadruplex inhibited the Cas12a activity, which, in turn, prevented the cleavage of the ssDNA probe around AuNPs. Subsequently, ssDNA-AuNPs flowed into the paper-based microfluidic chip and were captured by the immobilized nanoflowers, resulting in a corresponding color change. Reprinted with permission from [110]. Copyright 2023 Elsevier B.V.

The heavy metal ions in food safety risk detection include lead, mercury, silver, cadmium, chromium, etc. Current rapid biosensor detection technologies transform heavy metal ion concentration into nucleic acid signals mainly through DNAzymes corresponding to metal ions. Similarly, CRISPR/Cas-based nucleic acid biosensors also adopt the same strategy. Chen et al. proposed a lead ion (Pb^2+^) detection method by combining DNAzyme and CRISPR/Cas12a. In the detection of Pb^2+^, the minimum detection limit was 0.48 nM. The method’s benefits include simplicity, high sensitivity, and the use of portable, 3D-printing-assist equipment [106]. Similarly, Li et al. developed a versatile CRISPR-Cas12a platform to detect Pb^2+^ with a minimum detection limit of 0.053 nM. The difference was that the DNAzymes were modified on the surface of magnetic beads [107]. To further enhance the sensitivity, Wen et al. designed a dual-functional DNAzyme combined with the CRISPR/Cas12a system to develop a fluorescence biosensor for the field detection of Pb^2+^. The results showed that Pb^2+^ could be detected with the fluorescence change, and the LOD was as low as 1 pg/L, demonstrating high sensitivity (Figure 7A) [108]. Apart from heavy metals, sodium ions (Na^+^) are of concern as core nutrients. Excessive intake of Na^+^ also affects human health. Xiong et al. developed a CRISPR-Cas12a biosensor for point-of-care diagnostics Na^+^. Na+ detection could be completed in 15 min, with a detection limit of 0.1 nM [111].

In addition to metal-induced cleavage activity, Pb^2+^ can induce the folding of G-rich sequences into G-quadruplex structures with higher stability. Yu et al. first found that the Pb^2+^-induced G-quadruplex could resist trans-cleavage by CRISPR-Cas12a. Then, a fluorescence biosensor was constructed to detect Pb^2+^, with a minimum detection limit of 2.6 nM. The biosensor had the advantages of high stability, high sensitivity, and low cost [109]. Based on this, Zhang et al. constructed a paper-based microfluidic biosensor for the portable detection of Pb^2+^. The detection of Pb^2+^ could be achieved by analyzing R/G values using SmartIons APP, with the lowest detection limit of 18.3 nM. The paper-based microfluidic biosensors showed great potential for the on-site detection of Pb^2+^, given the low cost, ease of customization, and multi-channel nature (Figure 7B) [110].

### 5.6. Detection of Pesticides and Veterinary Drugs

**Figure 8:**
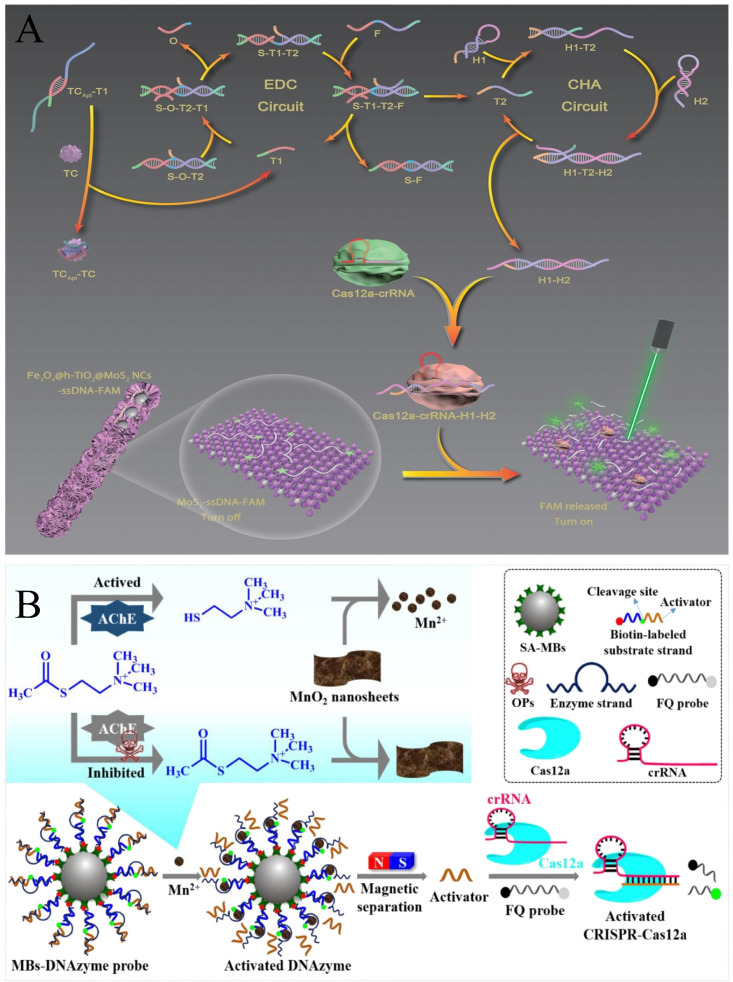
Principal diagram of pesticide and veterinary drug detection by CRISPR/Cas-based nucleic acid biosensors. (**A**) Schematic illustration of TCApt-EDC-CHA-Cas12a sensing for the detection of tetracycline. In the presence of tetracycline, the release of the complementary strand from the aptamer triggered the cascade amplification circuit, which, in turn, activated the activity of Cas12a to cleave FAM-ssDNA to restore fluorescence. Reprinted with permission from [117]. Copyright 2023 Wiley-VCH GmbH. (**B**) Schematic illustration of the AChE-mediated fluorescence biosensor for the detection of OPs. In the absence of OPs, TCh containing reducing sulfhydryl groups was generated, which induced the degradation of MnO_2_ nanosheets and produced Mn^2+^ to cleavage the Mn^2+^-dependent DNAzyme. Then, the released short DNA activated the activity of Cas12a to cleavage ssDNA probes, resulting in a change in fluorescence. Reprinted with permission from [114]. Copyright 2022 Elsevier Ltd.

Pesticides and veterinary drug residue in food mainly originate from the use of pesticides and veterinary drugs in the agricultural production process. Pesticides are used to control crop pests and diseases and veterinary drugs are used to treat animal diseases. These chemicals are potentially harmful to human health as they may remain in crops and animal products during use and thus enter the food chain. In general, the detection of pesticides and veterinary drugs using CRISPR/Cas-based nucleic acid biosensors requires the assistance of aptamers. Li et al. combined CRISPR/Cas12a with luminescent molecules to develop a novel detection platform for pesticide testing. Without the need for nucleic acid amplification, the CRISPR system was able to specifically detect the activator DNA complementary to the aptamer, and the LOD could reach 2.7 pM, which was suitable for trace acetamiprid detection [112]. Similarly, Yee et al. developed a fluorescent aptasensor based on the CRISPR-Cas12a effector and polystyrene microspheres to detect tetracycline, showing detection limits of 0.1 μM [116]. With the help of nucleic acid amplification, the detection sensitivity could be further enhanced. Yan et al. fabricated a multifunctional logic sensing system based on the CRISPR/Cas12a system and signal amplification circuits for the detection of two pesticides. The dual detection results in acetamiprid (ACE) and atrazine (ATR) showed high target specificity, with detection limits as low as 2.5 pM and 0.2 pM for ACE and ATR, respectively [113]. In addition, the catalytic activity of nanomaterials could also be applied for detection. Lv et al. combined the CRISPR/Cas12a system with the EDC-CHA network circuits and multifunctional Fe_3_O_4_@hollow-TiO_2_@MoS_2_ nanochains to develop a fluorescence sensing platform applied to the highly sensitive detection of tetracycline, with a limit of detection of 0.384 pg/mL (Figure 8A) [117].

OPs can phosphorylate acetylcholinesterase (AChE), leading to inhibition of the activity of AChE. AChE catalyzes the hydrolysis of ATCh to thiocholine (TCh). Therefore, a series of innovative ideas have been devised that utilize targets to hinder enzyme activity in cascade reactions. Fu et al. proposed an AChE-mediated fluorescence biosensor based on AChE and the CRISPR-Cas12a system for organophosphorus pesticide (OP) detection. The limits of detection for paraoxon, dichlorvos, and demeton were 270, 406, and 218 pg/mL, respectively (Figure 8B) [114]. It was found that Mn^2+^ significantly accelerated the cis- and *trans*-cleavage activity of CRISPR-Cas12a. By combining the catalytic activity of AChE with the Mn^2+^-activated CRISPR-Cas12a (MACC) system, Tian et al. developed a fluorescence biosensor for detecting carbaryl. As an inhibitor of AChE, carbaryl was successfully detected through the change of fluorescence, with a detection limit of 29 nM [115].

### 5.7. Detection of Others

In addition to the above categories of risk factors, there are other types of risk factors, such as viruses, parasites, illegal additives, and small molecules. Wang et al. selected cestode-derived miRNA let-7-5p as a biomarker for analyzing cestodes and established an RCA-assisted CRISPR/Cas9 assay to achieve early diagnosis of the parasite. The biosensor exhibited excellent diagnostic performance of let-7-5p and enabled detection at a limit as low as 10 aM [118]. Cas14-based platforms also have good potential for the detection of food hazards. Zhao. et al. combined the CRISPR/Cas14a system with G-quadruplex DNAzyme and a paper-based microfluidic device to develop a novel colorimetric biosensor, which was applied to the detection of ASFV. The biosensor provides excellent specificity as a two-nucleotide mismatched ASFV sequence and high sensitivity, with a limit detection as low as 5 copies/μL, indicating its potential in actual clinical diagnosis [119]. Ma et al. reported an aptasensor combining CRISPR/Cas14a1 with Exo III, which was applied to the detection of melamine. According to the change in fluorescence intensity, the sensitive detection of melamine was realized, with a limit of detection of 3.2 nM [120].

## 6. Conclusions and Prospects

The discovery of the CRISPR/Cas system has revolutionized molecular diagnostics, and the Cas protein has also become an effective biometric component of biosensing systems. CRISPR/Cas-based nucleic acid biosensors have also shown excellent capabilities and potential for detecting pathogenic bacteria, toxins, heavy metals, and residues. Compared with other technologies, this technology is low-cost, less time-consuming, and does not require specialized personnel or special equipment. These features have significantly expanded the use of CRISPR/Cas-based nucleic acid biosensors. In this review, we focused specifically on the design ideas for CRISPR/Cas-based nucleic acid biosensors. Among them, type V and type VI effector proteins have the greatest potential for biosensor construction because they can non-specifically cleave differently labeled reporter molecules to provide signal output. Meanwhile, the construction strategies of different types of nucleic acid amplification technologies and different types of signal output systems in CRISPR/Cas-based nucleic acid biosensors were highlighted and summarized, and the applications in the field of food analysis were presented. As mentioned above, these CRISPR sensors can provide rapid diagnoses of food risks. However, as encouraging as these breakthroughs are, it is worth noting that CRISPR/Cas-based nucleic acid biosensors still face a lot of challenges in future development and have a lot of room for improvement.

First, CRISPR/Cas-based nucleic acid detection technology mainly consists of two steps: nucleic acid amplification and Cas protein-mediated signal detection. For targets with low concentrations, nucleic acid amplification can significantly amplify original signals and promote the maximization of the activity of Cas protein. However, it still has some disadvantages, such as the need for primer design and amplification equipment. For other nucleic acid analyses, while significantly increasing the sensitivity of the assay, it also increases the complexity of the assay and, most importantly, may lead to cross-contamination and result in false positive results. Although many existing techniques can overcome this drawback, they increase operational complexity, so it is necessary to further explore more effective methods in the future. In addition, for some NAATs, the reaction temperature is not suitable for subsequent Cas activity, which must be analyzed in a two-step process. This is both cumbersome and increases the risk of contamination. Therefore, combining these two steps into one is an important development direction for the future.

Second, CRISPR/Cas-based nucleic acid biosensors can make early warning screens for food safety. However, the accuracy of detection of target molecules is confounded by the complexity of the food matrix. More importantly, the complexity of food samples can cause trace detection to become more difficult. Therefore, the development of advanced sample pretreatment techniques to remove matrix components that interfere with detection is also important in the assay. If CRISPR/Cas-based nucleic acid biosensors are combined with advanced sample preprocessing techniques to build an integrated analysis platform, this can significantly simplify analytical procedures, shorten the time to process samples, and reduce assay costs. In addition, food matrices interfere with non-nucleic acid targets mainly in terms of recognition. In contrast, CRISPR/Cas-based nucleic acid biosensors require the assistance of functional nucleic acids for the detection of non-nucleic acid targets. However, the ability of functional nucleic acids to recognize targets is affected by matrix interference, which affects subsequent assay accuracy and sensitivity. This requires screening to optimize functional nucleic acids for the recognition of elements with higher affinity.

Third, the output systems of CRISPR/Cas-based nucleic acid biosensors include fluorescence, colorimetric, and electrical signals, among which, fluorescence and colorimetric analyses can achieve naked-eye detection only at a qualitative level. Even portable devices, such as lateral flow strips and microfluidic chips, can only reach qualitative or semi-quantitative levels. While electrochemical signals can provide higher biosensor detection sensitivity, they are dependent on an electrochemical platform for electrochemical signal analysis. Although the sensitivity has been upgraded through the adaptation of different signal output strategies, there has been no improvement in signal analysis equipment. Therefore, under the premise of ensuring detection sensitivity, improving the portability of detection equipment is also the focus of future research. More portable equipment should be developed in the future, supplemented by 3D-printing technology, intelligent analysis systems, etc., to build more portable and intelligent CRISPR/Cas-based nucleic acid biosensors.

Fourth, in food safety testing applications, CRISPR/Cas-based nucleic acid biosensors realize the rapid and accurate analysis of pathogenic bacteria and toxins, heavy metals, and biological components. However, food safety problems are often caused by a variety of factors, and, therefore, the development of multiple detection capabilities of biosensors should also be a focus of future research. The multiplexing of assays in a single reaction volume is challenging due to interference between multiple reaction steps, which can affect assay sensitivity and specificity. This problem is mainly due to the random nature of *trans*-cleavage. The activation of Cas protein (Cas12, Cas13, or Cas14) activity by any of the target DNAs triggers the cleavage of all ssDNA/ssRNA probes. So far, most CRISPR/Cas-based nucleic acid biosensors realize multiple detection through physical separation. A major downside of this method is that the separation relies on an independently designed companion device. In addition, multiplexed detection has also been achieved in a single system through the selectivity of different Cas proteins. However, the detection throughput of such a multiplex approach is limited by the number of different Cas proteins with different nucleotide preferences, which, in turn, is limited by current biotechnology. Therefore, the performance of Cas proteins should be deeply explored in the future, such as seeking breakthroughs in cleavage performance and realizing the simplicity of multiplexed assays.

## Figures and Tables

**Table 1:** Key features of common CRISPR/Cas systems.

	Cas9	Cas12a (Cpf1)	Cas12b (C2c1)	Cas13a (C2c2)	Cas14 (Cas12f)
Classification	Class II Type II	Class II Type V	Class II Type V	Class II Type VI	Class II Type V
Nuclease Domains	HNH, RuvC	RuvC only	RuvC only	HEPN × 2	RuvC only
Guide RNA	crRNA, tracrRNA	crRNA only	crRNA, tracrRNA	crRNA only	crRNA, tracrRNA
Spacer Length	18–24 nt	16–30 nt (commonly, 20 nt)	20 nt	22–28 nt	20–25 nt
Cis-Cleavage Target	dsDNA with PAM	dsDNA with PAM or ssDNA	dsDNA with PAM or ssDNA	ssRNA	ssDNA
PAM Sequence	NGG	(T)TTN	TTN	No PAM but PFS	/
Cis-Cleavage Site	3 bases 5′ of the PAM	dsDNA: ~14/18–23 bp 3′ of the PAM ssDNA: ~22 nt from the first 3′ guide-complementary base	dsDNA: ~17–24 bp 3′ of the PAM ssDNA: /	Many cleavage sites, U and A	Beyond the guide-complementary region
*Trans*-Cleavage Target	/	ssDNA	ssDNA	ssRNA	ssDNA

**Table 2:** The application of CRISPR/Cas-based nucleic acid biosensor for food analysis.

Targets	Recognition Elements	Effector Protein	Signal Amplification	Signal Reporter	Signal Output	Sensitivity	Results Evaluation	References
*Staphylococcus aureus*	Genome	Cas12a	PCR	F/Q-ssDNA	Fluorescence	10^3^ CFU/mL	Can be finished within 2 H, but requires pre-culture and genome extraction.	[85]
*Staphylococcus aureus*	Genome	Cas12a	RAA	QDs-SA-Bio-ssDNA	Colorimetric lateral flow strip	5.4 × 10^2^ CFU/mL	RAA shortens the assay time but requires pre-culture and genome extraction.	[86]
*Vibrio parahaemolyticus*	Genome	Cas12a	LAMP	G4 ssDNA	Colorimetric	6.1 × 10^2^ CFU/mL	Visual analysis requires pre-culture and genome extraction.	[87]
*Burkholderia pseudomallei*	Genome	Cas14a	/	PtPd@PCN-224-ssDNA	Electrochemical	12.8 aM of DNA	Ultra-sensitive analysis of DNA.	[88]
*Staphylococcus aureus* *Pseudomonas aeruginosa*	Genome	Cas13a and Cas13b	Multiple RPA	F/Q-poly U F/Q-poly A	Fluorescence	As low as the attomolar range	Multiple analyses but poor generalizability.	[48]
*Escherichia coli* O157:H7	Aptamer	Cas12a	RCA	MB-ssDNA	Electrochemical	10 CFU/mL	Avoids genome extraction and improves assay sensitivity.	[89]
*Salmonella Typhimurium*	Aptamer	Cas12a	/	F/Q-ssDNA	Fluorescence	6.31 × 10^3^ CFU/mL	Easy to operate but lacking in sensitivity.	[90]
GMO species	P-35S and *T*-nos	Cas12a	RPA	F/Q-ssDNA	Fluorescence	10 copies	Simple to operate and finished within 15 min at room temperature.	[91]
GMO species	P-35S and *T*-nos	Cas12a	RPA	FeAuG-MB and G4 ssDNA	SERS	At the pM level	Significant increase in sensitivity with the aid of SERS.	[92]
GMO species	P-35S	Cas12a	/	F/Q-ssDNA	Fluorescence	0.1%	Direct detection of short fragments using multiple crRNAs expands application in further processed products.	[93]
GM maize	P-35S, GUS, and *T*-nos	Cas12a	Multiplex LAMP	F/Q-ssDNA	Fluorescence	100 aM	Multiple detections but realized by system separation.	[94]
GM soybeans	P-35S and *lectin*	Cas12a	Dual LAMP	F/Q-ssDNA	Fluorescence	0.1%	Multiple detections but realized by system separation.	[95]
Pork, beef, and duck	Genome	Cas12a	RPA	F/Q-ssDNA	Fluorescence	100, 10, and 10 copies	Simple to operate and finished within 15 min at room temperature.	[91]
Chicken, duck, beef, pork, and lamb	Genome	Cas12a	RPA	F/Q-ssDNA and AuNPs	Fluorescence and colorimetric lateral flow strip	1 × 100 copies/μL	Suitable for on-site identification of large quantities of meat.	[96]
Goat milk	Genome	Cas12a	LAMP	PB-ssDNA	SERS	224 aM	Ultra-sensitive detection with the help of Raman signals.	[97]
Pufferfish	Genome	Cas12a	PCR	NiCo_2_O_4_ NCs@Au-ABEI-ssDNA	Electrochemical	0.1%	Provides a simple, low-cost, and sensitive approach to trace pufferfish adulteration.	[98]
Pork	Genome	Cas12a	RPA	F/Q-ssDNA	Fluorescence	10^−3^ ng	Simplified genome extraction process and the detection could be completed within 30 min.	[99]
Pig, chicken, duck, and lamb	Genome	Cas12a	RAA	F/Q-ssDNA	Fluorescence	0.1%	Integration of amplification analysis with the help of centrifugal microfluidic chip.	[100]
Ochratoxin A	Aptamer	Cas12a	/	F/Q-ssDNA	Fluorescence	171 fg/mL	Lower price and easier preparation.	[101]
Ochratoxin A	Aptamer	Cas12a	/	UCNP-ssDNA-Fe_3_O_4_	Upconversion fluorescence	1.5641 ng/mL	Rapid and stable, but slightly lacking in sensitivity.	[102]
Ochratoxin A	Aptamer	Cas12a	Exo III-assisted recycling system	Fc-ssDNA	Electrochemical	0.74 fg/mL	Enhances sensitivity through nucleic acid amplification.	[103]
Lipopolysaccharide	Aptamer and Mg^2+^-independent DNAzyme	Cas12a	/	F/Q-ssDNA	Fluorescence	7.31 fg/mL	Ingenious DNA enzyme-walking nanomachines with high sensitivity.	[104]
Aflatoxin M_1_	Aptamer	Cas12a	RCA	AuNPs-ssDNA and 4-nitrophenol	Colorimetric	0.05 ng/L	RCA adds operational complexity without a significant increase in sensitivity.	[105]
Pb^2+^	Pb^2+^-independent DNAzyme	Cas12a	/	F/Q-ssDNA	Fluorescence	0.48 nM	Simplicity and high sensitivity; also with designed 3D-printing equipment to assist fluorescence analysis.	[106]
Pb^2+^	Pb^2+^-independent DNAzyme	Cas12a	/	F/Q-ssDNA	Fluorescence	0.053 nM	Reduced system interference and increased sensitivity with the help of magnetic separation.	[107]
Pb^2+^	MB-DNAzyme/AuNP	Cas12a	/	F/Q-ssDNA	Fluorescence	1 pg/L	Ultrasensitive and high-throughput detection.	[108]
Pb^2+^	Na^+^/Pb^2+^-induced G-quadruplex	Cas12a	/	F/Q-G4 ssDNA	Fluorescence	2.6 nM	First provides a new method for Pb^2+^ detection based on induced G-quadruplex inhibition on CRISPR-Cas12a *trans*-cleavage.	[109]
Pb^2+^	Pb^2+^-induced G-quadruplex	Cas12a	/	AuNPs-ssDNA	Colorimetric	18.3 nM	A chip-scale paper microfluidic smart analysis system that has great potential for the in situ detection of Pb^2+^.	[110]
Na^+^	Na^+^-independent DNAzyme	Cas12a	/	F/Q-ssDNA	Fluorescence	0.10 mM	Simple and fast inspection workflow—two steps in less than 15 min.	[111]
Acetamiprid	Aptamer	Cas12a	/	Fc-ssDNA	Electrochemical	2.7 pM	Designed PTCA-COF shows strong and stable ECL emission signals for enhanced detection sensitivity.	[112]
Acetamiprid and atrazine	Aptamer	Cas12a	Toehold-mediated strand displacement reaction	F/Q-ssDNA	Fluorescence	2.5 pM 0.2 pM	With high sensitivity, the realization of multiple analyses relies on logic gate design.	[113]
Paraoxon, dichlorvos, and demeton	Acetylcholinesterase and Mn^2+^ dependence of the DNAzyme	Cas12a	/	F/Q-ssDNA	Fluorescence	270 pg/mL, 406 pg/mL, 218 pg/mL	Great versatility for detecting OPs.	[114]
Carbaryl	Acetylcholinesterase and Mn^2+^ dependence of the DNAzyme	Cas12a	/	F/Q-ssDNA	Fluorescence	29 nM	Can be extended for rapid screening of pesticide residues.	[115]
Tetracycline	Aptamer	Cas12a	/	Polystyrene microsphere/Q-ssDNA	Fluorescence	0.1 μM	The problems of being time-consuming and costly, giving false positive signals, and having a complex primer design and difficult optimization phase are solved.	[116]
Tetracycline	Aptamer	Cas12a	Cascade amplification circuits	FAM-ssDNA-Fe_3_O_4_@hollow-TiO_2_@MoS_2_	Fluorescence	0.384 pg/mL	The introduction of nanomaterials significantly improves detection sensitivity.	[117]
Parasite	Cestode-derived miRNA let-7-5p	Cas9	RCA	RCA products complement F/Q-ssDNA	Fluorescence	10 aM	RCA enables signal amplification but increases operational complexity for Cas9.	[118]
African swine fever virus	Genome	Cas14a	PCR	G4 ssDNA	Colorimetric	5 copies/μL	Paper-based microfluidic device significantly improves assay portability.	[119]
Melamine	Aptamer	Cas14a1	/	F/Q-ssDNA	Fluorescence	3.2 nM	Enhancement of affinity by aptamer modification to improve assay specificity and sensitivity.	[120]

## Data Availability

No new data were created or analyzed in this study. Data sharing is not applicable to this article.
